# Extraction and Analysis of Respiratory Motion Using Wearable Inertial Sensor System during Trunk Motion

**DOI:** 10.3390/s17122932

**Published:** 2017-12-17

**Authors:** Apoorva Gaidhani, Kee S. Moon, Yusuf Ozturk, Sung Q. Lee, Woosub Youm

**Affiliations:** 1 Department of Mechanical Engineering, San Diego State University, 5500 Campanile Drive, San Diego, CA 92182, USA; apoorva2501@gmail.com; 2 Department of Electrical and Computer Engineering, San Diego State University, 5500 Campanile Drive, San Diego, CA 92182, USA; yozturk@mail.sdsu.edu; 3 Electronics and Telecommunications Research Institute, ICT, 218 Gajeong-ro, Yuseong-gu, Daejeon 34129, Korea; hermann@etri.re.kr (S.Q.L.); wsyoum@etri.re.kr (W.Y.)

**Keywords:** biomedical signal processing, wearable biomedical sensors, medical equipment, wireless sensor network, E-healthcare

## Abstract

Respiratory activity is an essential vital sign of life that can indicate changes in typical breathing patterns and irregular body functions such as asthma and panic attacks. Many times, there is a need to monitor breathing activity while performing day-to-day functions such as standing, bending, trunk stretching or during yoga exercises. A single IMU (inertial measurement unit) can be used in measuring respiratory motion; however, breathing motion data may be influenced by a body trunk movement that occurs while recording respiratory activity. This research employs a pair of wireless, wearable IMU sensors custom-made by the Department of Electrical Engineering at San Diego State University. After appropriate sensor placement for data collection, this research applies principles of robotics, using the Denavit-Hartenberg convention, to extract relative angular motion between the two sensors. One of the obtained relative joint angles in the “Sagittal” plane predominantly yields respiratory activity. An improvised version of the proposed method and wearable, wireless sensors can be suitable to extract respiratory information while performing sports or exercises, as they do not restrict body motion or the choice of location to gather data.

## 1. Introduction

The accurate measurement and monitoring of physiological parameters plays an essential role in a broad range of applications in the field of healthcare, psycho-physiological examinations, and sports training. Respiration rate as a physiological parameter is usually measured when the subject is at rest, counting the number of breaths per minute by counting the number of times the chest expands. Respiration rate as a vital sign is affected by exercise. Exercise increases the rate at which energy is needed. It increases the need for oxygen in the body. During exercise, the heart speeds up to pump extra food and oxygen to the muscles. Breathing speeds up to get more oxygen and to get rid of more carbon dioxide. Calorie expenditure during exercise can be estimated by measuring the ventilation and the exchange of oxygen and carbon dioxide by the body. There is substantial evidence that an abnormal respiratory rate is a predictor of potentially serious clinical events. Monitoring respiration rate is therefore of interest not only for assessment of medical conditions, but also monitoring the intensity of exercise and calculating the calorie expenditure. Monitoring respiration rate during exercise specifically when the body trunk is moving is the focus of this article. 

It has been reported in a recent study conducted by Harvard Health School [1] that gentle stretching and strengthening of back and abdominal muscles can reduce lower back pain. Such exercises build strong and flexible muscles that are less prone to injury, pain, strains, and sprains [1]. Trunk stretching exercises are performed to maintain a flexible trunk to make it less vulnerable to injury and sprains. These exercises often restore the normal range of motion of the trunk. Stretching is also known to improve balance by preventing falls and impart relief from arthritis, knee pain, and so forth [2,3,4]. Stretching becomes an essential and integral part of life. Further, stretching and yoga can consume a significant number of calories depending upon the BMI (body mass index) and duration of exercise [5]. Trunk motion information can be used to derive an accurate estimation of calories burned while exercising. Thus, real-time monitoring of exercise motions including lateral flexion (sideward bending), forward bending (flexion), backward bending (extension) and rotation (twisting) would be valuable. 

Time-dependent assessment of respiratory motion is useful in exercise or clinical applications. Koch and Dietzel provided a unique 6 × 6 sensor array for curvature sensing in the format of a thin, flexible polyimide foil for respiratory monitoring of premature infants by directly attaching it to the skin [6]. Also, a respiratory plethysmography is employed to determine the changes in chest volume to monitor breathing using a flexible sensor [7]. In a study performed by Gollee and Chen, a single IMU (inertial measurement unit) placed on the abdomen was used to detect breathing motion when the body is motionless [8]. However, when the trunk was in motion, the movement was reflected as respiratory action. Measuring respiration with a single IMU when the body moves is difficult, as the body trunk motion obscures the breathing motion. Thus, there is a need to decompose the respiration motion from the overall fused, unified or integrated motion irrespective of heterogeneity or complexity of the trunk movement. This paper proposes a novel method to resolve this issue using a two IMU sensor systems to extract the respiratory motion. The effect of coughing on the respiratory action has also been studied in this research. The proposed sensors system may also help to detect coughing episodes to quantify exercise-induced asthma [9].

The research put forth in this article makes use of a pair of wireless IMU sensors placed at specific locations on the human trunk so that respiratory motion can be extracted while performing trunk motion. These sensors are custom-made by the authors at San Diego State University [10]. The sensor is small, wearable, and wireless with a built-in (on-chip) recharging module [10]. This study applies the principles of robotic kinematics, using the Denavit-Hartenberg convention [11] to extract relative motion [12] between the two IMU sensors. The method decomposes the motions experienced by the two IMUs into trunk movements and breathing actions. The concept used here spans several daily applications, apart from diagnosing abnormalities, such as monitoring respiration while exercising. Further analysis of the breathing signal can yield respiration rate, which can be used to calculate calories burned indirectly.

## 2. Materials and Methods

**Figure 1:**
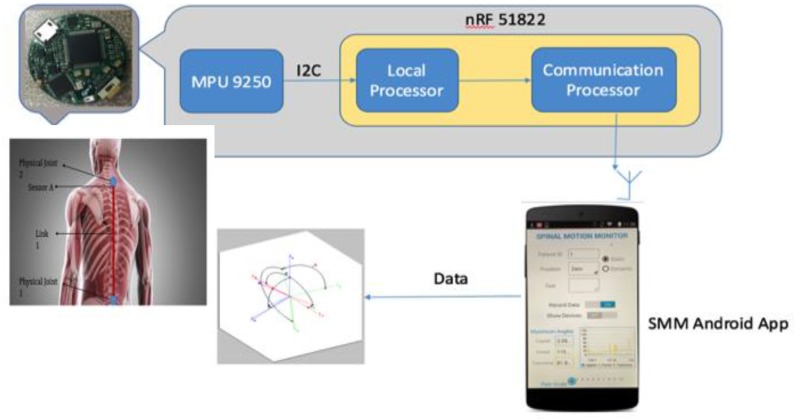
San Diego State University wearable wireless motion sensor network.

The material requirement of this research is a pair of IMU sensors to create a network of joints and links that can mimic a three-dimensional (3-D) robot model of a portion of the human trunk. The wireless motion sensor developed in this study uses a nine-axis motion processor from InvenSense (MPU9250, InvenSense, San Jose, CA, USA) and a system-on-chip (SoC) from Nordic Semiconductor (nRF51822, Nordic Semiconductor, Oslo, Norway). Each wearable sensor node (3 cm in diameter and 0.2 cm thick) is wirelessly connected to an Android application (Figure 1). Biocompatible adhesive tapes are used to accurately secure these sensors in the designated areas in the trunk. An Android device acts as host receiver to the data transmitted by these sensors [10]. The Android device must be Bluetooth 4.0 enabled and must have the data collection or sensor configuring host application installed to allow collection of data in the receiver [10]. A MATLAB program was developed for required mathematical calculations.

A crucial part of this research was the selection of the location for sensors placement. Three criteria were followed in choosing the location: (1) IMU 1 (Sensor A) should majorly detect trunk motion (with minimal or no respiratory motion); (2) IMU 2 (Sensor B) should experience both trunk and respiratory action, and; (3) the trunk motion experienced by both the sensors must be of the same amplitude (or range). As there are two regions on the trunk that can provide information on respiratory motion, namely, the chest and abdomen, the range of motion of trunk at these levels was analyzed. 

The trunk motion was considered to be analogous to vertebral movement, and hence the range of motion of spine was studied at chest and abdomen levels. The chest region falls in front of the upper thoracic curve, and the abdomen falls in front of the lumbar curve (after thoracic), which is two of the five regions of the spine/vertebra. The range of motion of spine at both these curves is different depending on the movement performed. In the case of flexion, extension and lateral flexion (sideward sway), the lumbar vertebral region moves more than the thoracic region, causing the chest to move more than the abdomen. 

**Figure 2:**
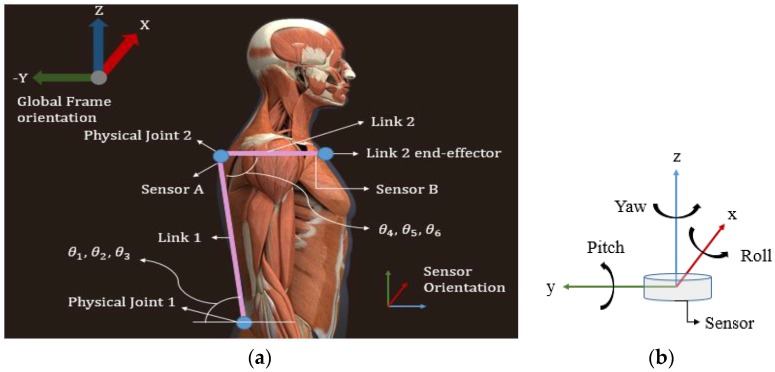
Sensor placement: (**a**) Shows location of joints of robot model along with placement of sensors. Both the physical joints are imaginary. Physical Joint I is located at the hip (hip joint), and Physical Joint II is located on the back (posterior side), just below the neck. Each physical joint has 3-DOF (degree-of-freedom) (three joints are combined to form each Physical Joint) imparting the robot system a total of 6-DOF. Links 1 and 2 are also imaginary and connect Physical Joint I to Physical Joint II, and Physical Joint II to Link 2 end-effector, respectively. Sensor A is placed at the end of Link 1 just before the Physical Joint II and, thus, it gives the orientation of Link 1 with respect to the global frame. Sensor B is placed at the end of Link 2 just before the Link 2 end-effector, and it gives the orientation of Link 2 with respect to the global frame. $\theta_{1},\theta_{2},\theta_{3}$ are the angles that Link 1 makes with the base frame (Frame 0) of the robot model that will be introduced ahead. $\theta_{4},\theta_{5},\theta_{6}$ are the angles that Link 2 makes with Link 1 and are found to provide the respiratory information; (**b**) The orientation of both sensors: ‘Roll’ is the rotation around “x-axis,” “pitch” around “y-axis” and “yaw” around “z-axis” of the sensor coordinates. All rotations follow anticlockwise-positive convention.

Based on these criteria, the sensors are placed on the chest and the posterior side of the chest. Figure 2 shows this sensor placement in standing position/posture. The orientation of both the sensors is maintained equally. The reason for this arrangement is the presence of external intercostal muscles under the rib cage, which provides maximum force during respiration [13]. As a result, these muscles serve as a good indication of breathing.

### Kinematic Equations and Mathematical Components

The data obtained from an IMU sensor is converted to Euler angles (more specifically, Tait-Bryan angles) [14]. Here, *ϕ* is roll, which portrays rotation around the local/sensor x-axis, *θ* is pitch denoting rotation around the y-axis, and *ψ* is yaw representing the rotation around the z-axis of a sensor (as seen in Figure 2b). Each quaternion (with four quantities) gives a set of Euler angles (having three quantities). These Euler angels are substituted into the following matrix in the sequence roll-pitch-yaw to obtain a rotation matrix [14].

(1) $$R\left(\phi ,\;\theta ,\;\psi \right)=\left[\begin{array}{ccc} c_{\theta }c_{\phi } & s_{\psi }s_{\theta }c_{\phi }-c_{\psi }s_{\phi } & c_{\psi }s_{\theta }c_{\phi }+s_{\psi }s_{\phi }\\ c_{\theta }s_{\phi } & s_{\psi }s_{\theta }s_{\phi }+c_{\psi }c_{\phi } & c_{\psi }s_{\theta }s_{\phi }-s_{\psi }c_{\phi }\\ -s_{\theta } & s_{\psi }c_{\theta } & c_{\psi }c_{\theta } \end{array}\right],$$

This matrix corresponds to ZYX rotation sequence of Tait-Bryan angles [14], where “*c*” stands for cosine and “*s*” stands for sine of the respective Euler angle. The Euler angles and rotation matrices are found for both the sensors separately; these give the orientation of each sensor with respect to the global coordinate frame. 

Homogeneous transformation matrices are 4 × 4 matrices containing a (3 × 3) rotational matrix in the upper left corner, which gives the orientation information, a (3 × 1) column vector in the upper right corner that gives the positional (or translational) information. Thus, a homogeneous matrix provides the “pose”—position and orientation information. 

**Figure 3:**
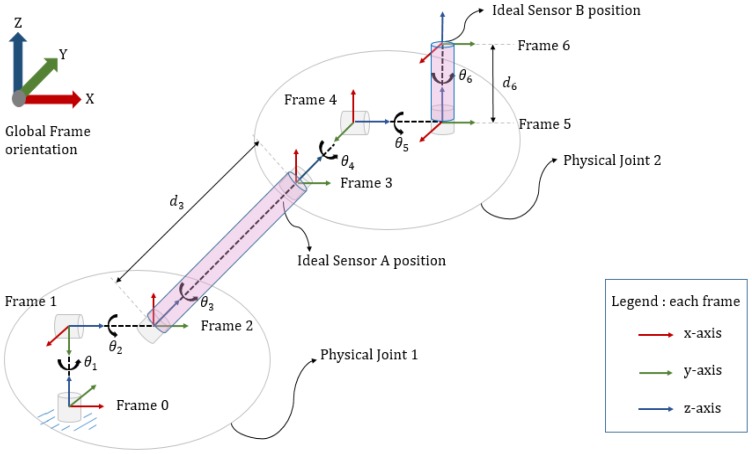
The 3-D geometry of a two-joint robot model with each joint having 3-DOF. Note that the origins of coordinate Frames 0, 1 and 2 coincide collectively, forming Physical Joint I, which has 3-DOF. The origins of Frame 3, 4 and 5 coincide to form Physical Joint II having 3-DOF.

Figure 3 shows the Denavit-Hartenberg (D-H) Convention [11] to assign right-handed frames to links connecting two joints. The D-H algorithm is used to derive forward kinematics [11] to find the position and orientation of end-effector of a robot when all the joint angles are known. The 3-D robot geometry consists of two physical-joints each having three degree-of-freedom (DOF). Thus, imparting the entire system a total of 6-DOF. The origins of Frame 0, 1 and 2 coincide to form a fused Physical Joint I. Similarly, the origins of Frame 3, 4 and 5 correspond to produce a fused Physical Joint II. All the theta angles in the model follow the anticlockwise-positive convention. Following the D-H Convention, link parameters or D-H parameters [11] are derived, as shown in Table 1. We consider $d_{3}$ and $d_{6}$ to be 43 cm and 6.12 cm, respectively, from the study of Ratnovsky and Elad [13]. $\alpha_{i}$ is an angle about common normal, from old z-axis to new z-axis. However, the parameter is well known in Denavit-Hartenberg convention. Currently, this research mainly concentrates on the orientation aspect of the two sensors or links of the devised 3-D robot model.

The four parameters of each link give a homogeneous transformation matrix $A_{i}$. Thus, six homogeneous transformation matrices are obtained. $T_{n}^{0}$ = $A_{1}$…$A_{n}$ gives the position and orientation information of robot’s end-effector (Frame *n*) in base frame (Frame 0), that is, the transformation or mapping of Frame *n* in Frame 0. $T_{n}^{0}$ is of the form of a homogeneous transformation matrix. As per D-H convention law, every $A_{i}$ can be represented as a product of four basic transformations [11] as shown below:

(2) $$A_{i}=Rot_{z,\theta_{i}}\;Trans_{z,d_{i}}\;Trans_{x,a_{i}}\;Rot_{x,a_{i}},$$

(3) $$A_{i}=[\begin{array}{cccc} c_{\theta_{i}} & -s_{\theta_{i}} & 0 & 0\\ s_{\theta_{i}} & c_{\theta_{i}} & 0 & 0\\ 0 & 0 & 1 & 0\\ 0 & 0 & 0 & 1 \end{array}][\begin{array}{cccc} 1 & 0 & 0 & 0\\ 0 & 1 & 0 & 0\\ 0 & 0 & 1 & d_{i}\\ 0 & 0 & 0 & 1 \end{array}][\begin{array}{cccc} 1 & 0 & 0 & a_{i}\\ 0 & 1 & 0 & 0\\ 0 & 0 & 1 & 0\\ 0 & 0 & 0 & 1 \end{array}][\begin{array}{cccc} 1 & 0 & 0 & 0\\ 0 & c_{\alpha_{i}} & -s_{\alpha_{i}} & 0\\ 0 & s_{\alpha_{i}} & c_{\alpha_{i}} & 0\\ 0 & 0 & 0 & 1 \end{array}],$$

(4) $$A_{i}=\left[\begin{array}{cccc} c_{\theta_{i}} & -s_{\theta_{i}}c_{\alpha_{i}} & s_{\theta_{i}}s_{\alpha_{i}} & a_{i}c_{\theta_{i}}\\ s_{\theta_{i}} & c_{\theta_{i}}c_{\alpha_{i}} & -c_{\theta_{i}}s_{\alpha_{i}} & a_{i}s_{\theta_{i}}\\ 0 & s_{\alpha_{i}} & c_{\alpha_{i}} & d_{i}\\ 0 & 0 & 0 & 1 \end{array}\right],$$

Here, “*s*” stands for sine and “*c*” stands for cosine of the respective angles. Equation (4) is the general form of homogeneous transformation matrix that varies with each link based on the D-H parameters of that link. Here, “*s*” stands for sine and “*c*” stands for cosine of the respective angles. Since the model distinctly consists of two physical links and joints, each having three rotational joints, the number of transformation matrices can be reduced from six to two, as follows:

(5) $$T_{3}^{0}=A_{1}^{0}\;A_{2}^{1}\;A_{3}^{2},$$

(6) $$T_{3}^{0}=[\begin{array}{cccc} c_{1}c_{2}c_{3}-s_{1}s_{3} & -c_{1}c_{2}s_{3}-s_{1}c_{3} & c_{1}s_{2} & d_{3}\left(c_{1}s_{2}\right)\\ s_{1}c_{2}c_{3}+c_{1}s_{3} & -s_{1}c_{2}s_{3}+c_{1}c_{3} & s_{1}s_{2} & d_{3}\left(s_{1}s_{2}\right)\\ -s_{2}c_{3} & s_{2}s_{3} & c_{2} & d_{3}\left(c_{2}\right)\\ 0 & 0 & 0 & 1 \end{array}],$$

(7) $$T_{6}^{3}=A_{4}^{3}\;A_{5}^{4}\;A_{6}^{5},$$

(8) $$T_{6}^{3}=[\begin{array}{cccc} c_{4}c_{5}c_{6}-s_{4}s_{6} & -c_{4}c_{5}s_{6}-s_{4}c_{6} & c_{4}s_{5} & d_{6}\left(c_{4}s_{5}\right)\\ s_{4}c_{5}c_{6}+c_{4}s_{6} & -s_{4}c_{5}s_{6}+c_{4}c_{6} & s_{4}s_{5} & d_{6}\left(s_{4}s_{5}\right)\\ -s_{5}c_{6} & s_{5}c_{6} & c_{5} & d_{6}\left(c_{5}\right)\\ 0 & 0 & 0 & 1 \end{array}],$$

where $c_{1}$, $c_{2}$, $c_{3}$, $c_{4}$, $c_{5}$, $c_{6}$ stand for cos ($\theta_{1}$), cos ($\theta_{2}$), cos ($\theta_{3}$), cos ($\theta_{4}$), cos ($\theta_{5}$), cos ($\theta_{6}$) and $s_{1}$, $s_{2}$, $s_{3}$, $s_{4}$, $s_{5}$, $s_{6}$ are sin $\left(\theta_{1}\right)$, sin ($\theta_{2}$), sin ($\theta_{3}$), sin $\left(\theta_{4}\right)$, sin ($\theta_{5}$), sin ($\theta_{6}$), respectively. Here, $T_{3}^{0}$ is the mapping of Frame 3 in Frame 0 and $T_{6}^{3}$ is mapping of Frame 6 in Frame 3. The global homogeneous transformation matrix $T_{0}^{G}$ is assumed to be an Identity matrix. As in this research, only the rotational part of the 4 × 4 homogeneous transformation matrix is considered, as per the model geometry and theoretical knowledge, rotation matrix obtained from Equation (1) for both the sensors A and B are equivalent to the rotational part of $T_{3}^{0}$ and $T_{6}^{0}$, or analogously $T_{3}^{G}$ and $T_{6}^{G},$ since $T_{0}^{G}$ is an identity matrix, that is:

(9) $$R_{3\times 3}\;(A)\equiv R_{3\times 3}\;(T_{3}^{0})\equiv R_{3\times 3}\;(T_{3}^{G}),$$

(10) $$R_{3\times 3}\;(B)\equiv R_{3\times 3}\;(T_{6\;\left(original\right)}^{0})\equiv R_{3\times 3}\;(T_{6}^{G}),$$

The forward kinematic equation to obtain the end-effector (Frame 6) orientation in/with respect to base frame (Frame 0) is given as follows:

(11) $$T_{6}^{G}=T_{0}^{G}*T_{3}^{0}*T_{6}^{3},$$

Rearranging the above equation to get orientation of sensor B with respect to sensors A or equivalently the orientation of Frame 6 with respect to Frame 3:

(12) $$T_{link21}=T_{6}^{3}={\left(T_{3}^{0}\right)}^{-1}*{\left(T_{0}^{G}\right)}^{-1}*T_{6}^{0},$$

where $T_{6}^{0}$ is the initialized version of $T_{6\;\left(original\right)}^{0}$. The initialization of rotation matrix obtained from sensor B is done in order to perfectly align sensor B ($T_{6}^{0}$) with sensor A ($T_{3}^{0}$) in the mid-sagittal plane. To initialize $T_{3\;\left(original\right)}^{0}$ and $T_{6\;\left(original\right)}^{0}$, the following operations as described in Equations (13) and (14) are performed as follows:

(13) $$T_{3}^{0}=\;T_{3\;\left(original\right)}^{0}*T_{3\;\left(inv\right)}^{0},$$

(14) $$T_{6}^{0}=\;T_{6\;\left(original\right)}^{0}*\;T_{6\;\left(inv\right)}^{0},$$

Next, inverse kinematic equations [11] are used to find out all robot joint angles when the end-effector orientation (rotation matrix) is known. Hence, joint angles are extracted from rotation matrices (orientation information) $T_{3}^{0}$ and $T_{6}^{3}$; where both are known, $T_{3}^{0}$ is directly obtained from sensor A and $T_{6}^{3}$ is obtained from the mathematical calculation described in Equation (12), which gives the relative orientation [11] of sensor B (Frame 6) with respect to sensor A (Frame 3). First, by applying inverse kinematics to $T_{3}^{0}$, joint angles $\theta_{1}$, $\theta_{2}$, $\theta_{3}$ that Link 1 makes with respect to robot model (Frame 0) are obtained.

(15) $$r_{33}=c_{2},\;\;\theta_{2}=\cos^{-1}(r_{33}),$$

(16) $$r_{31}=-s_{2}c_{3},\;\;\theta_{3}=\;\cos^{-1}\left(\frac{-r_{31}}{s_{2}}\right),$$

(17) $$r_{13}=c_{1}s_{2},\;\;\theta_{1}=\cos^{-1}\left(\frac{r_{13}}{s_{2}}\right),$$

Next, by applying inverse kinematics [11] to $T_{6}^{3}$, relative joint angles $\theta_{4}$, $\theta_{5}$, $\theta_{6}$ that Link 2 makes with Link 1 are obtained. Similarly, we extract:

(18) $$r_{33}=c_{5},\;\;\theta_{5}=\cos^{-1}(r_{33}),$$

(19) $$r_{31}=-s_{5}c_{6},\;\;\theta_{6}=\cos^{-1}\left(\frac{-r_{31}}{s_{5}}\right),$$

(20) $$r_{13}=c_{4}s_{5},\;\;\theta_{4}=\cos^{-1}\left(\frac{r_{13}}{s_{5}}\right),$$

## 3. Experimental Results

**Figure 4:**
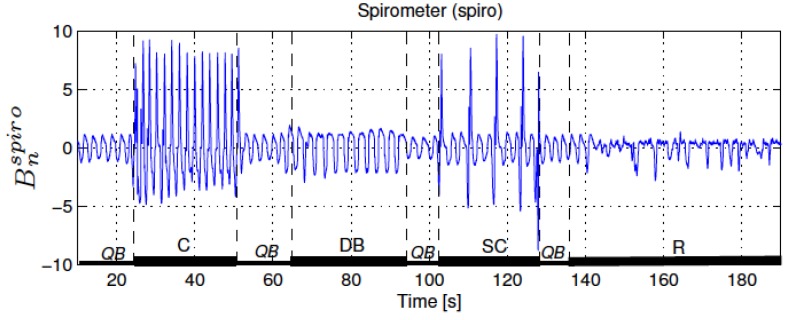
A reference measurement of the respiratory activity, the airflow at the mouth and nose was measured using a spirometer [8].

This section provides a discussion of the experimental results obtained in this research. Figure 4 presents a reference measurement of the respiratory activity; the airflow at the mouth and nose was measured using a spirometer that is a standard instrumentation for respiratory monitoring.

An experimental protocol was followed while collecting the data in the described sensor placement. All the experiments were performed in the standing posture. The protocols include trunk motion during performing normal breathing interspersed with coughing episodes to mimic abnormal breathing. Coughing induces pattern variation in respiratory sinusoidal wave and creates specific patterns in breathing activity graph. The experiments performed are summarized in Table 2. Experiment I is the standard case showing normal breathing pattern with bend-extend trunk motion. Experiment II includes a single cough pattern that is mimicked after normal breaths. 

### 3.1. Bend-Extend Trunk-Motion (Experiment I)

**Figure 5:**
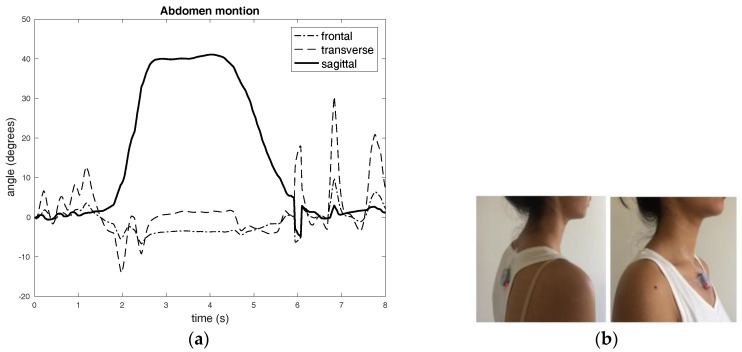

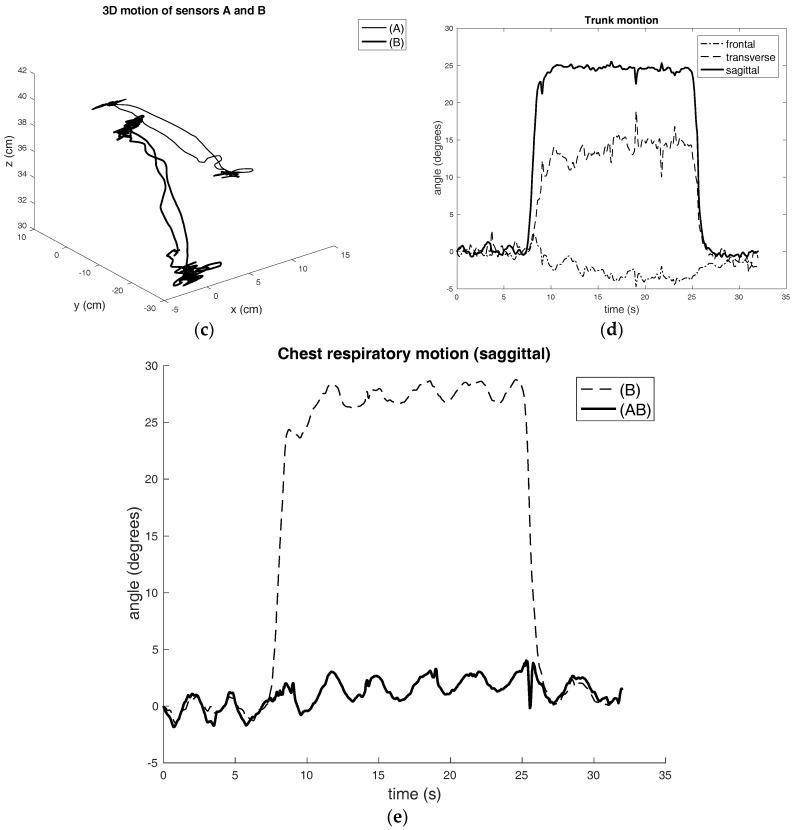
(**a**) Normal breathing accompanied by bending-extending trunk-motion: It was measured using single IMU (inertial measurement unit) located on the abdomen. The abdomen joint angle shows bending motion of trunk in the “Sagittal” plane. Other abdomen angles have minimum deviations showing some noisy breathing signals. The sensor motions show the trunk motion has been reflected in the precision of the respiration signals. (**b**) The pictures show the two IMU sensor placements on the trunk in coherence with that shown in Figure 2a. (**c**) The 3-D Cartesian motions of sensors A and B: The sensor motions clearly show the bending motion has been reflected in the graph. (**d**) Normal breathing accompanied by bending-extending trunk-motion: The hip joint angles show bending motion of trunk in the “Sagittal” plane. Some of the bending trunk motion has been reflected in the “Transverse” plane. The hip joint in the “Frontal” plane shows negligible deviation, as this experiment did not involve bending motion. (**e**) Normal breathing accompanied by bending-extending trunk-motion: The breathing signals and the “Sagittal” motions of sensors A and B: The “Sagittal” plane motion of sensor B shows some breathing signals with a significant bending motions. The very clear breathing motions of ribcage joint angles from sensors A and B (decomposed chest motion with respect to dorsal chest position) in the “Sagittal” plane. Sensor B signals show bend-extend motion (downward-upward deviation, respectively).

Figure 5 shows the experiments involving normal breathing with bend-extend trunk motion.

### 3.2. Cough-Motion

**Figure 6:**
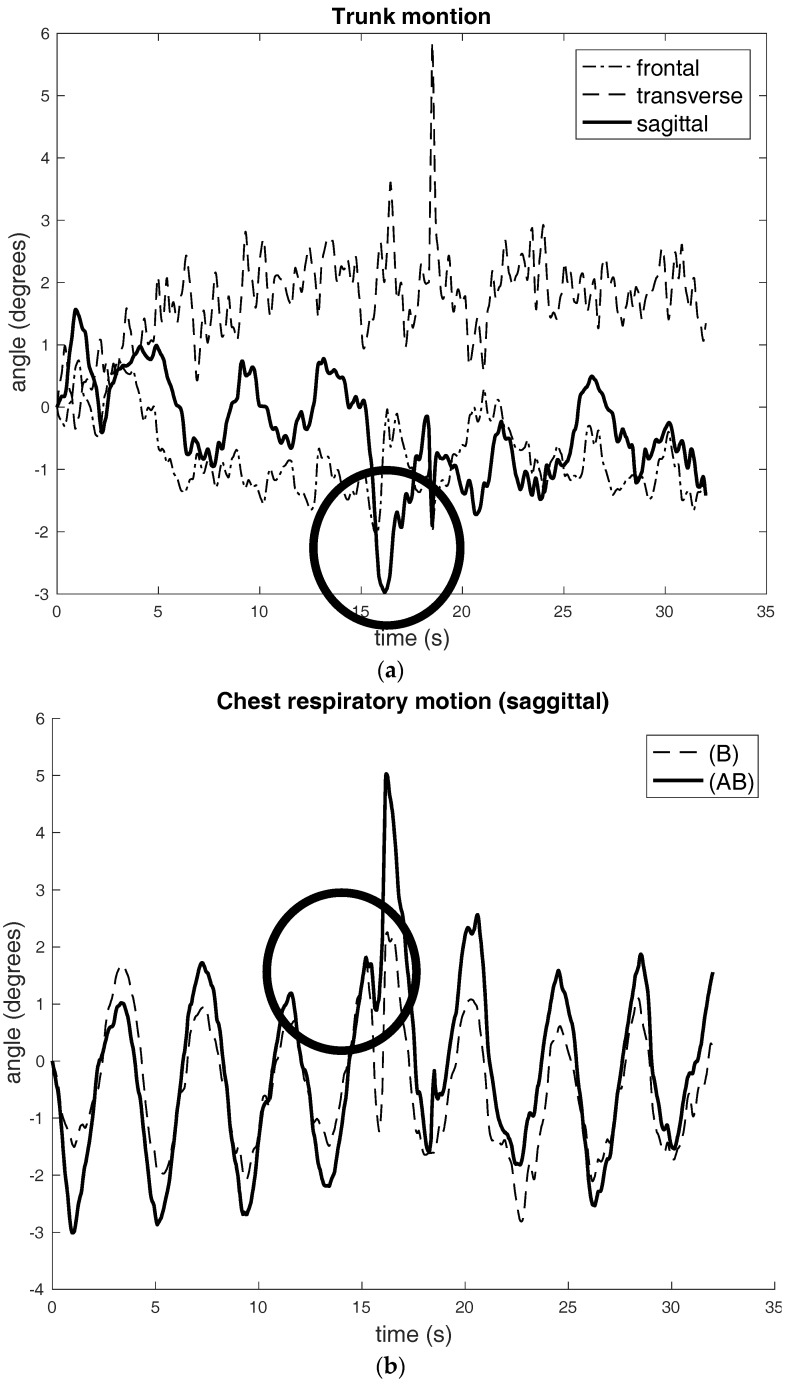
Abnormal breathing (single-cough) with no trunk motion: (**a**) Single-cough breathing with no trunk motion: The very clear coughing motions of trunk shows in the “Sagittal” plane; (**b**) The single coughing can be seen as short and sharp negative spikes, which have been indicated by a circle.

Figure 6 shows an experiment involved abnormal breathing (a single cough) with no trunk motion.

## 4. Discussion and Conclusions

This research aimed at eliminating the influence of body motion on breathing motion. The proposed mathematical algorithm and sensor placement are successful in extracting the breathing signals from a fused set of data containing trunk and breathing motion. In this paper, the ribcage joint angles from sensors A and B (decomposed chest motion with respect to dorsal chest position) in the “Sagittal” plane shows breathing signals. It is worth noting that corresponding Euler angles of the two sensors must be similar in pattern as well as magnitude to ensure precise and accurate extraction of respiratory motion in one of the relative joint angles. Dissimilarities between the two are reflected in the relative angles, and therefore degrade respiratory information. 

The proposed sensor placement gives promising results. The results also show that human trunk does always perfectly sway while performing sway motion. Instead, it may tend to show some additional movement in bending or twisting simultaneously. The IMU device used for this study is small, light-weight, wearable and wireless with a long battery life. This design makes it an excellent choice for long-term data collection while performing mild trunk-stretching exercises like yoga or other body activities without causing hindrance or restriction of body motion. These findings can be used in future research to invent technologies with contemporary requirements that include devices using wearable IMUs to record real-time breathing motion during day-to-day activities, especially useful for elderly and for people suffering from panic attacks, Parkinson’s disease, Alzheimer’s, and so on, whose movements and breathing need to be monitored. This technology can also be advanced further for the real-time detection of asthma attacks, which are often accompanied by shortness of breath and coughing. Additionally, apnea (central sleep apnea) and SIDS (sudden infant death syndrome) diagnosis and detection can be made more accessible in real time by using an improvised version of this technology. 

Overall, this technique can be extended to several domains to find use in many future applications. Though this research works well with data from one individual, it should be tested on a larger sample size to make it a generalized study, which can be universally accepted. Data can also be collected using commercially available IMUs to check the validity of the data obtained in this study. In future, more IMUs can be recruited to form a network of IMUs to model the desired body region to be studied. Overall, this research proves to be a promising step towards using and further enhancing the presented concept. In summary, this paper presents a novel method to approach the problem of breath detection by fusing the information deriving from two different IMUs placed on the anterior and posterior side of the chest (sternum and spine). Further, the method can be improved by including one more IMU placed on the abdomen to cover the abdominal breathing, and also provide a method for globally evaluating the breath activity.

## Figures and Tables

**Table 1:** Denavit-Hartenberg (D-H) parameters of 3-D model shown in Figure 3. The parameter with an asterisk (*) mark denotes that it is variable.

Link	$a_{i}$	$d_{i}$	$\alpha_{i}$	$\theta_{i}$
1	0	$d_{1}=0$	$-90^{{}^{\circ}}$	$\theta_{1}^{*}$
2	0	$d_{2}=0$	$90^{{}^{\circ}}$	$\theta_{2}^{*}$
3	0	$d_{3}=43\;\mathrm{cm}$	$0^{{}^{\circ}}$	$\theta_{3}^{*}$
4	0	$d_{4}=0$	$-90^{{}^{\circ}}$	$\theta_{4}^{*}$
5	0	$d_{5}=0$	$90^{{}^{\circ}}$	$\theta_{5}^{*}$
6	0	$d_{6}=6.12\;\mathrm{cm}$	$0^{{}^{\circ}}$	$\theta_{6}^{*}$

**Table 2:** Overview of Experiments.

Experiment	Sensor Placement	Breathing Style/Pattern	Trunk Motion
I	Dorsal Chest & Chest	Normal	Bend-extend
II	Dorsal Chest & Chest	Abnormal single-cough	None
